# Sexual Transmission of HCV in Heterologous Monogamous Spouses

**DOI:** 10.1155/2014/140640

**Published:** 2014-11-24

**Authors:** Mona M. Rafik, Yehia El Shazly, Amal A. Abbas, Walid Abd Elhady, Dina Ragab, Dina AlShennawy

**Affiliations:** ^1^Clinical and Chemical Pathology Department, Faculty of Medicine, Ain Shams University, Cairo 11566, Egypt; ^2^General Medicine & Gastroenterology Department, Faculty of Medicine, Ain Shams University, Abbassia Square, Cairo 11566, Egypt

## Abstract

We screened for evidence of HCV infection in healthy heterologous monogamous spouses of chronic HCV patients and studied the relation with various risk factors. A cross-sectional study of fifty healthy monogamous heterosexual spouses of HCV-positive index cases was carried out. All participants were HBV and HIV negative. The association with various risk factors was studied. Five spouses (10%) showed evidence of HCV infection. Two partners were positive for HCV antibody alone (4%) and 3 for antibody and HCV PCR (6%). No association was found between HCV infection and various sociodemographic parameters with the exception of older age categories. Intraspousal transmission of HCV may be an important source of spread of HCV infection. The reservoir of HCV-infected individuals in Egypt is sizable, and sexual transmission of HCV may contribute to the total burden of infection in Egypt.

## 1. Introduction

The extent to which HCV infection is associated with sexual exposure has been debated extensively. The 2008 Egyptian Demographic and Health Survey [1] estimated that 15% of people aged 15–59 years in Egypt have anti-HCV antibodies and 10% have chronic HCV infection. However, this high figure is related to past transmission events and is not representative of the HCV transmission occurring in Egypt. Sexual transmission of HCV may contribute to the total burden of infection in Egypt. The objectives of this study were to detect the risk for sexual transmission of HCV infection from chronically infected subjects to their long-term monogamous heterosexual partners (50 spouses) and to identify various sexual practices associated with that risk. Spouses were carefully selected to exclude risk factors and participants were HBV (HBsAg, HBcAb, and HBsAb) and HIV antibody negative. All participants were tested for liver enzymes, HCV specific antibodies (Ortho Clinical Diagnostics, New Jersey, USA), and HCV RNA assay with real-time PCR using Qiagen extraction kit and Brilliant HCV QRT-PCR for Stratagene's Mx3000P. Five spouses showed evidence of HCV infection, two were positive for HCV antibody alone (4%) and three were positive for HCV antibody and HCV PCR (6%). No association was found between the HCV infection status and various selected risk factors such as sex, age, duration of marriage, frequency of sexual intercourse, history of sexually transmitted diseases, and other risk factors for HCV. The only association detected was the age category where both positivity of HCV antibody and HCV PCR were associated with the older age category (>60 y). As regards the direction of transmission, an insignificant difference between male and female spouses of chronic HCV-4 infected patients was detected. In this study the prevalence of HCV in the heterosexual spoused is 10% which may seem to be lower than the general population. However, our patient population was carefully selected so that sexual intercourse would be the major risk factor. One possible explanation is that a higher cell-mediated immune response against HCV in repeatedly exposed sexual partners would partially protect them against infection. The detection of HCV-specific cellular immune responses in seronegative sexual partners of HCV patients has been documented [2, 3].

There are some limitations in this study. An important limitation is failure to analyze the sequence of nucleotides of the HCV genome. The detection of homology in the nucleotide sequences would have been a strong evidence of a common source of infection but it would not clarify the direction of the infection, nor the responsible risk factors. The major limitation of our study may be the relatively small number of participants.

## 2. Conclusion

Our study results raise the possibility that HCV is sexually transmitted between spouses in Egypt. This confirms the need to screen all people in the so-called high risk groups for HCV infection. Due to the ongoing high incidence of HCV in Egypt, further research is needed to identify the exact routes of transmission and the associated risk factors so that preventive measures can be instituted.
